# Volatile-mediated plant interactions: an innovative approach to cultivar mixture selection for enhanced pest resilience

**DOI:** 10.3389/fpls.2025.1550678

**Published:** 2025-04-08

**Authors:** Dimitrije Markovic, Gaëtan Seimandi-Corda, Vili Harizanova, Atanaska Stoeva, Sari Himanen, Stephanie Saussure, Andja Radonjic, Gordana Đurić, Ivana Lalićević, Sokha Kheam, Merlin Rensing, Jannicke Gallinger, Samantha M. Cook, Velemir Ninkovic

**Affiliations:** ^1^ Department of Ecology, Swedish University of Agricultural Sciences, Uppsala, Sweden; ^2^ Faculty of Agriculture, University of Banja Luka, Banja Luka, Bosnia and Herzegovina; ^3^ Protecting Crops and the Environment, Rothamsted Research, Harpenden, Hertfordshire, United Kingdom; ^4^ French National Research Institute for Agriculture, Food and Environment (INRAE), National Polytechnic Institute of Toulouse (INPT), PURPAN Engineering School (EI PURPAN), AGroécologie - Innovations – TeRritoires (AGIR), Toulouse, France; ^5^ Agricultural University of Plovdiv, Plovdiv, Bulgaria; ^6^ Natural Resources Institute Finland (Luke), Jokioinen, Finland; ^7^ Faculty of Agriculture, University of Belgrade, Belgrade, Serbia; ^8^ Institute for Plant Protection and Environment, Belgrade, Serbia; ^9^ Department of Biology, Faculty of Science, Royal University of Phnom Penh, Phnom Penh, Cambodia; ^10^ Federal Research Centre for Cultivated Plants, Institute for Plant Protection in Fruit Crops and Viticulture, Dossenheim, Germany

**Keywords:** plant-plant communication, aphid infestation, sustainable pest management, within species plant diversity, winter wheat, spring barley, aphid population development, grain yield

## Abstract

Mixing different cultivars has been recognized as a promising strategy for the reduction of pest pressure and the enhancement of crop performance. However, this applies only in specific combinations, creating a need to select cultivars that interact synergistically in mixtures. We propose a trait-based laboratory method to identify complementary pairs of cereal cultivars based on their ability to prime one another’s defense response through volatile organic compounds (VOCs). In this study, we screened 25 locally-grown cultivars from six European countries to assess their responsiveness to volatile priming under controlled conditions. The tested cultivars exhibited three primary types of volatile interactions: no interaction, one-way interaction (where one cultivar responded to volatiles from another) and two-way interaction (where both cultivars reciprocally responded). Subsequently, the efficacy of these cultivar pairs was evaluated over a three-year period in field trials where aphid infestation, natural enemy abundance and plant traits (height, number of plants per 1-meter, Thousand Grain Weight (TGW) and yield) were assessed. Field trials results demonstrated that only specific cultivar mixtures led to a significant reduction in aphid infestation, indicating a robust genetic and environmental interaction. Mixtures in which both cultivars exhibited two-way interaction under controlled conditions, demonstrated reductions in aphid abundance in comparison to monoculture controls. In contrast, the abundance of natural enemies was not significantly affected by cultivar mixtures, and there were no notable changes in plant traits. We propose that the strategic pairing of cultivars, which actively engage in volatile interactions in the laboratory, can effectively reduce aphid pressure in the field without compromising plant traits or crop yield, thereby reducing reliance on chemical control. Given the role of aphids as vectors of economically significant viruses, reducing their population could also limit the spread of plant diseases in the field. This approach underscores the importance of understanding plant interactions at a chemical level to optimize cultivar pairing and develop sustainable pest management strategies.

## Introduction

An increase in biodiversity is of paramount importance for the enhancement of ecological stability, the optimization of resource utilization, and the ultimate improvement of productivity and resilience in both wild and cultivated plant systems (Tilman et al., 2014; Dainese et al., 2019). These ecosystem services which include pollination, natural pest control, soil fertility and water regulation, are essential for maintaining agricultural productivity, ecological balance and long-term environmental resilience (Power, 2010). In agricultural systems, the increase in intraspecific diversity through the introduction of cultivar mixtures has emerged as an effective strategy for simultaneously enhancing multiple ecosystem services (Snyder et al., 2020). The integration of cultivars with different functional traits such as plant height can lead to more efficient resource partitioning by mixtures, which in turn increases crop productivity and yield stability (Creissen et al., 2016; Borg et al., 2018; Döring and Elsalahy, 2022). While some studies reported increased yield stability in cultivar mixtures (Borg et al., 2018; Wuest et al., 2021; Kong et al., 2023; Huang et al., 2024), others have found limited or no benefits compared to single cultivars (Mansion-Vaquié et al., 2019; Grettenberger and Tooker, 2020). These discrepancies suggest that cultivar mixtures do not consistently demonstrate superior performance in terms of yield when compared to their individual components.

Beyond enhancing crop productivity, cultivar mixtures have been shown to suppress pest insects and diseases while simultaneously fostering populations of natural enemies (Tooker and Frank, 2012; Koricheva and Hayes, 2018; Wan et al., 2022). However, recent studies showed that the effectiveness of cultivar mixtures in reducing pest pressure is contingent upon the specific cultivar combinations employed, highlighting the context-dependent nature of these interactions (Tooker and Frank, 2012; Dahlin et al., 2018, 2020). The precise mechanisms by which cultivar mixtures reduce pest populations remain unclear, as these effects are often limited to specific cultivar combinations. This underscores the importance of selecting the ‘right kind of diversity’ within the field to achieve positive interactions and desired outcomes (Shoffner and Tooker, 2013). Several mechanisms have been proposed to explain the natural pest control provided by cultivar mixtures. Bottom up control relates to the production of secondary compounds that deter the herbivores or dilution effects that decrease the apparent abundance of host plants, thereby reducing their detectability by pests (Borg et al., 2018). Top-down control, postulates that increased plant genetic diversity attracts a diversity of natural enemies which in turn reduce pest populations (Barbosa et al., 2009). This is because natural enemies rely on plant chemical cues when foraging, interacting with plants when selecting habitats, and ultimately use these cues to locate and capture herbivores (Grettenberger and Tooker, 2020; Kheam et al., 2024b). Significant increases in natural enemies, such as ladybirds, have been observed in certain soybean cultivar mixtures at specific plant growth stages (Kheam et al., 2024b). Additionally, ladybirds also showed preference to specific combination of barley cultivars over their pure stands prior to the aphids arrival in the field (Ninkovic et al., 2011). These findings suggest that an increased genetic diversity within specific cultivar mixtures at a particular growth stage can enhance the plants’ attractiveness to natural enemies.

One mechanism underlying this effect involves volatile organic compounds (VOCs), which are released by all plants but undergo qualitative and quantitative changes following herbivory (Hare, 2011). These herbivore induced plant volatiles (HIPV) serve as airborne signals, priming neighboring plants to activate their defense mechanisms more rapidly and effectively against potential threats, such as herbivore attacks (Heil and Karban, 2010; Mauch-Mani et al., 2017). Defense priming is an adaptive, low-cost strategy that activates initial defense responses, enabling plants to mount faster, stronger, and more sustained response to subsequent pest infestation (Martinez-Medina et al., 2016). Even undamaged or uninfected plants can influence the defense mechanisms of neighboring plants via constitutively released VOCs, reducing their susceptibility to future infestations (Ninkovic et al., 2019). For instance, exposure of barley cultivar Salome to volatiles from another cultivar, Anakin, induced higher emission of trans‐β‐ocimene, which disrupted aphid olfactory preferences and decreased their relative growth rate and intrinsic rate of increase (Kheam et al., 2023, 2024a). Recent studies have shown that plants not only respond to their abiotic environment but also to the identity of their neighbors, as expressed by the specific profile of VOCs they release (Ninkovic et al., 2021; Kheam et al., 2023, 2024a).

This insight provides the possibility of selecting cultivar mixtures based on the capacity of one cultivar to prime a defense response in another, thereby preparing them for potential herbivore attacks. A deeper understanding of the chemical interactions between undamaged plants via constitutively released VOCs and their impact on herbivore behavior is imperative for elucidating the inconsistent outcomes observed in field trials and also for enhancing the predictability of pest suppression in cultivar mixtures. To ensure the effective implementation of this strategy, it is essential to select cultivars on the basis of their distinctive functional traits, such as VOC emission, which may prime defense responses in other cultivars. This approach ensures that the combined traits will result in synergistic effect, thereby optimizing the ecological benefits of cultivar diversity. The considerable variability, spatial requirements and maintenance demands associated with field trials present a significant challenge in the screening of large numbers of cultivar mixtures. We argue that there is significant untapped potential in the design of optimal cultivar mixtures which can be realized through the development of new laboratory method for identification of complementary cultivars based on their interactions via volatiles.

The aim of this study was to determine whether pairs of winter wheat or spring barley cultivars, selected in the laboratory on the basis of their capacity to prime defenses in neighbors via constitutively VOCs, could effectively reduce aphid populations under field conditions. We hypothesized that cultivar pairs that demonstrated a reduction in aphid acceptance under controlled laboratory conditions would also exhibit reduction in aphid infestation when grown as field mixtures. This hypothesis is supported by a recent study showing that VOC emissions from one specific barley cultivar can prime defenses in another neighboring cultivar, reducing relative growth rate and intrinsic rate of increase of bird cherry aphid – *Rhopalosiphum padi* L (Kheam et al., 2023). To validate laboratory findings in agricultural settings, we conducted field trials across six European countries. These trials aimed to determine whether VOCs mediated interactions observed in the lab persist under variable field conditions, whether their effects on aphid populations are predictable across environments, and how they influence plant traits and crop productivity. The incorporation of our laboratory screening method into the deliberate selection of cultivars for mixtures for specific environmental conditions is intended to facilitate the development and implementation of resilient cropping systems that maintain productivity while supporting the presence of natural enemies.

## Materials and methods

### Cultivar screening in controlled conditions

A total of 12 spring barley (*Hordeum vulgare* L.) from Bosnia and Herzegovina, Finland and Sweden, and 13 winter wheat (*Triticum aestivum* L.) cultivars from Bosnia and Herzegovina, Bulgaria, Serbia, and United Kingdom were selected for screening in controlled conditions (**Table 1**). Seeds of commercially available cultivars were sourced from local distributors in each country, while seeds of other cultivars were collected from experimental farm plots. The selection of cultivars for screening was based on differences in their height and growth pattern (fast or slow) traits (Essah and Stoskopf, 2002). The seeds of all selected cultivars were dispatched to the Department of Ecology at the Swedish University of Agricultural Sciences for laboratory screening. The effect of volatile interactions between cultivars on aphid plant acceptance was assessed before sowing in 2018 for winter wheat and in 2019 for spring barley.

**Table 1:** List of cereal (spring barley or winter wheat) cultivars selected for field experiments in six countries over four years (2019-2022) .

Type of cereal	Country	Season	Cultivar 1	Cultivar 2	Cultivar 3
Spring barley	Bosnia and Herzegovina	2019	Matej	Jaran	Scarlett
		2021	Matej	Jaran	Scarlett
		2022	Matej	Jaran	
	Finland	2020	Alvari	Toria	Vertti
		2021			
	Sweden	2019	Salome	Fairytale	Anakin
		2020	Salome	Fairytale	Anakin
		2021	Salome	Fairytale	Anakin
		2022	Salome	Fairytale	
Winter wheat	Bosnia and Herzegovina	2019/2020	Simonida	NS40S	NS Rani otkos
	Serbia	2019/2020	Simonida	NS40S	NS Rani otkos
		2020/2021	Simonida	NS40S	NS Rani otkos
		2021/2022		NS40S	NS Rani otkos
	Bulgaria	2019/2020	Enola	Apolon	Lazarka
		2020/2021	Enola	Apolon	Lazarka
		2021/2022	Enola	Apolon	
	Finland	2021/2022	Huima	Tuomas	
	United Kingdom	2019/2020	Claire	Hereward	Xi19
		2020/2021	Claire	Hereward	Xi19
		2021/2022	Claire	Hereward	

Prior to sowing, seeds were germinated on wet filter paper in Petri dishes for 24 hours at room temperature. Six seeds were sown per pot (9 × 9 × 7 cm) filled with P-soil (Hasselfors, Sweden) and maintained in a growth chamber for 7 days at 18–22 C°, 50–60% relative humidity and L16:D8 h photoperiod. At the one-leaf stage, seven days after sowing, plants of uniform size were selected and placed in the exposure system.

### Exposure system

The effects of volatile interactions between different cultivars on aphid acceptance on receiving plants were investigated following the exposure to VOCs emitted by neighboring plants (**Supplementary Figure S1**). The exposure of one cultivar to another was conducted using all cultivars from the same country, without pairing cultivars between countries, because the objective was to identify pairs of locally adapted cultivars. One cultivar was exposed to VOCs from another in twin Perspex cages (Kheam et al., 2023). Each cage is divided into two chambers: one ‘emitting’ chamber and one ‘receiving’ chamber (each 10 × 10 × 40 cm), connected by a circular opening (7 cm in diameter) in the partitioning wall. The system is ventilated by a pump that draws air into the “emitting” chamber, where the emitter plant is located. The air then flows through the opening into the “receiving” chamber, which houses the receiver plant, before being vented outside the room. The airflow within the system was maintained at the rate of 1.2 L/min (Markovic et al., 2019). To prevent any potential interactions via root exudates between the emitter and receiver plants, each individual pot containing six seedlings was placed in a Petri dish. Each cultivar was exposed to the volatile compounds of another cultivar for a period of five days. A receiving cultivar exposed to clean air was used as a control. After a five day exposure period, the receiving plants were subjected to an aphid acceptance test. Four out of six plants in receiving pots were randomly chosen for aphid plant acceptance tests. Each treatment (receiving cultivar exposed to VOCs released from another emitter cultivar) was represented by five replicates.

### Aphid plant acceptance test

The objective of this laboratory test was to identify pairs of cultivars that interact via VOCs and prime neighboring plants to effectively reduce aphid plant acceptance. The bird cherry-oat aphid (*Rhopalosiphum padi* L.) was used as a model insect because it is one of the first cereal aphids to colonize plants in the spring season. The aphids used in all experiments were reared on oat (*Avena sativa* L.) cultivar Belinda in separate growth chamber under the same conditions as described for the plants.

The bioassay setup consisted of a transparent 100-ml polystyrene tube (2.5 cm diameter and 25 cm in length). The second leaf of a single receiver plant was inserted into one end of the tube without detatchment from the main plant. The leaf was passed through a slit made in a plastic sponge that plugged the tube’s end. Ten wingless *R. padi* (second to fourth instars) were placed at the opposite end of the tube to the leaf, which was then sealed with nylon netting to prevent their escape. To prevent mechanical damage to the plants, the bioassay tube was attached to a wooden stick to provide support (**Supplementary Figure S2**). The number of aphids settled on the leaf was recorded after two hours, a sufficient time for aphids to settle and reach the phloem (Prado and Tjallingii, 1997). The leaf was then removed from the tube and two parameters were used to assess aphid plant acceptance. First, the leaf was gently shaken for approximately 10 seconds to dislodge any loosely attached aphids without affecting those that had truly settled. Second, following (Powell et al., 1993), remaining aphids on the leaf were observed closely. If the aphid’s body did not move and its antennae were held back without any movement, the aphid was recorded as having settled. Each cultivar combination was tested in 20 replicates.

### Field trials

Field trials were conducted in six countries covering different pedoclimatic conditions over three growing seasons from 2018/19 to 2021/22. A total of 14 trials were performed to test the effect of selected winter wheat or spring barley cultivar mixtures on aphid infestation, natural enemy abundance, plant traits and crop yield.

Small-scale plots measuring 60 m^2^ (6 ^m^ x 10 m) were arranged in a Latin square design (except in Sweden in 2019, 2020, and 2021 where plots were arranged in Random Complete Block Design (RCBD)). A one-meter gap without any vegetation was maintained as a barrier between the experimental plots, and the distance between the field trial and the surrounding crop was at least one meter. Six treatments were sown in each field trial, one of each of three cultivars sown in monoculture plots and each combination of two cultivars in a 50:50 mixture (three combinations in total). This resulted in a total of 36 plots with 18 sown with a single cultivar and 18 sown with each combination of two cultivars. In 2021-2022 season, only two cultivars were tested instead of three, and each of the three treatments (the two monoculture and the mixture) were replicated six times, leading to a total of 18 plots. The trials were conducted without insecticide application, adhering to standard agricultural practices adapted to each location.

### Insect monitoring

In each plot, three main cereal aphid species were identified at the species level: the bird cherry-oat aphid (*R. padi* L.), the rose-grain aphid (*Metopolophium dirhodum* Walker) and the English grain aphid (*Sitobion avenae* Fabricius). The number of aphids (wingless and winged aphids) was counted by carefully inspecting the leaves and stems of all plants encountered along three randomly selected 1-meter transects within each plot, positioned at least 0.5 meters from the plot edges (Ninkovic et al., 2003). For winter wheat, aphid incidence was monitored weekly throughout the autumn-winter period (October-December) and the spring-summer period (May-July). In spring barley, aphid infestation was monitored across the spring-summer period (May-July). Monitoring began after the initial detection of aphids in the field and continued until their abundance significantly decreased when the crop started to desiccate. On the same day as aphid monitoring, the natural enemies of aphids, specifically Coccinellidae (ladybirds) and Syrphidae (hoverflies) were also recorded per plot. Two observers walked slowly along edge of the plot and observed each half of the plot canopy and above it (Ninkovic et al., 2011). These observations took place between 11:00 and 14:00 with favorable weather conditions such as low wind speed and clear skies, whenever possible. To ensure comparability of the analyses, the abundance of the three cereal aphid species was summed per transect for each monitoring occasion. Similar variation was observed in the abundance of natural enemies, thus the number of Coccinellidae and Syrphidae were summed per plot for each monitoring occasion.

### Plant trait measurements

Prior to tillering, the number of emerged plants was counted along three 1-metre transects in each plot. The number of plants per transect was also assessed at the time of harvest, by uprooting and counting the number of plants.

Plant height was measured on twenty, randomly selected plants per plot, by measuring the distance from the soil surface to the end of the upper leaf sheath on each plant (Pérez-Harguindeguy et al., 2013) when plants had reached BBCH 40 (emergence of the flag leaf) (Lancashire et al., 1991).

At the time of harvest, grain samples were taken from each plot and used to estimate the yield (kg/ha) after standardizing the moisture levels across all plots. After drying the seeds in an oven, the Thousand Grain Weight (TGW) was measured. In Bulgaria, the yield and TGW were estimated by measuring the weight of the grains collected along three pooled 1-meter transects. In other countries, seeds were obtained directly from the combine harvester, whereas in the UK both methods were employed.

### Statistical analysis

Data collected from the aphid plant acceptance test conducted in controlled conditions were analyzed using the proportion of aphids accepted plants in each tube with a Generalized Mixed Model following a binomial distribution and a logit link. The pot with plants was regarded as a random factor. The fixed part of the model included Cultivar 1 (‘Inducing cultivar’), Cultivar 2 (‘Receiving cultivar’), and the interaction between these. Least square means of the model parameters were calculated and compared. The significances were assessed both in unadjusted form and using Bonferroni adjustment.

For the insect monitoring, the number of aphids and natural enemy occurrence varied between field trials, ranging from 3 to 12. To standardize the data available, only three sampling occasions around the peak abundance of aphids were used for the analyses of aphid abundance and of their natural enemies.

To assess the effect of cultivar mixtures on field measurements (aphid abundance, abundance of natural enemies, plant height, plant number, TGW and yield), we adopted an approach comparing data from plots with cultivar mixtures to an expected mixture, calculated using data from monoculture plots. This method is a common practice for studies on cultivar mixtures and is often applied to yield analysis (e.g., relative yield index) (Reiss and Drinkwater, 2018; Tratwal and Bocianowski, 2018). It enables testing of whether or not the observations in the mixture are different from that expected with a mixture of the two cultivars and if there is a synergetic effect of the mixture. The trials were arranged in a Latin square design, ensuring each treatment appeared only once per row and column. For each pair of cultivars used in the trial, the expected mixture value was estimated by calculating the mean of the data collected from their corresponding monoculture plots within the same row and column (e.g., four plots in total) (**Supplementary Figure S3**). When measurements were taken multiple times (counting aphids and natural enemies), this calculation was performed separately for each sampling date. The resulting mean provided the expected value in mixtures for a given pair of cultivars.

To test the effect of the cultivar mixture, the expected in value in the cultivar mixture was compared to the observed value using a linear mixed model. Each model explained the values by the type of value (observed or expected), and the pair of cultivars imbricated in the type of value. The column and the row of the design were always included as random effects. If measurements were collected on multiple dates, the date was also included as random factor. For the aphids and natural enemies, abundance was transformed using log(x+1) to improve the normality of the residuals. For the data collected in 2022, only one pair of cultivars was tested per trial therefore the effect of the pair of cultivars was not tested. If model fit was singular, the model was replaced by a linear model with the same variables, but the random effects were used as fixed terms. For the field trials sown as RCBD, a procedure equivalent to the Latin squares was applied except that only the sum of the values from a block was used instead of the row and column. In the models explaining data in RCBD trials, the row and column effects were replaced by a block effect.

Normality and heteroscedasticity of the model residuals were checked using the DHAMaA package (Hartig and Hartig, 2022). The effect of each fixed effect was tested using a Wald χ^2^ test or an F-test depending on whether the model used was mixed or not (function *Anova* package car (Fox et al., 2013). The effect of the mixture was estimated by extracting coefficient parameters from the model (function *standardize_parameters* package effect size (Ben-Shachar et al., 2020). The standard coefficient of the variable and the associated 95% confidence interval of the coefficient was used to identify if the effect of the cultivar mixture had a coefficient different from 0 for each pair of cultivars tested. If the boundaries of confidence interval of the coefficient were lower than 0, the value measured was lower in the mixture than expected based on monocultures, and on the other hand, if the boundaries were higher than 0, the value measured was higher in mixture than in monoculture (Boetzl et al., 2023). Following this approach, a standard coefficient was extracted to test the effect of the cultivar mixture for each cultivar mixture tested, in each year and country.

Based on the coefficients extracted from the models, a meta-analysis was run to test if a global effect of the cultivar mixture could be observed on the different types of data collected (aphid abundance, abundance of natural enemies, plant height, plant number, TGW and yield). This approach allows analysis of the data from multiple studies and integrates them to test the effect of a given variable. Meta-analyses are particularly appropriate to account for the heterogeneity of date from the field trials in term of design (here Latin Square and RCBD) and sampling which is the case in the present study (Stewart et al., 2008). The coefficient parameters extracted from the previous models were used as effect size and the standard error of the effect size was estimated based on the confidence interval using the formula:


$$SE=\frac{CI\;upper\;limit-CI\;lower\;limit}{3.92}$$

A multilevel meta-analytic model was then run for each type of data measured, as a variable to explain the measurements, and with a cofactor for the year, the country, and the crop species as random effects (rma.mv function, package metaphor (Viechtbauer, 2010)). A Wald χ^2^ tests was then run for each model. The *predict.rma* function (package metaphor (Viechtbauer, 2010)) was used to calculate the predictions and confidence intervals at 95% of the variable in each model.

Based on the aphid plant acceptance tests conducted in controlled conditions, cultivar pairs were assigned to three categories of response: no interaction, one-way interaction, and two-way interaction. Another multilevel meta-analytic model was then run for the field aphid data. The model then included as moderator the response category (no interaction, one-way interaction, and, two-way interaction) and as random factor the country, year, and crop species. The same procedure as previously described for the meta-analytic model was followed. All the statistics were conducted using R 4.3.1 (R Core Team, 2022).

## Results

### Cultivar screening in controlled laboratory conditions

The screening results allowed us to identify and characterize three main types of volatile interactions among the tested cultivars. ‘No interaction’ was inferred if the receiver cultivar after being exposed to VOCs from the emitting cultivar showed no change in aphid plant acceptance compared to the control. ‘One-way interaction’ was inferred if the receiver cultivar exhibited defense responses characterized by reduced aphid plant acceptance only when exposed to volatiles from a particular emitting cultivar. ‘Two-way interaction’ was inferred if the emitter VOCs induced defense in receiver cultivar, resulting in a reduced aphid acceptance and this defense priming was observed to occur reciprocally when the receiver was tested as the emitter.

A significant reduction in aphid plant acceptance was observed in receiver cultivars after exposure to volatiles released from some of the specific emitting cultivars compared to control plants exposed to clean air (**Table 2**). In the winter wheat cultivars, the pairs Enola+Lazarka (Bulgaria), NS40S+NS Rani otkos (Bosnia and Herzegovina, and Serbia), Hereward+Xi19 and Claire+Hereward (United Kingdom) demonstrated significant reduction in aphid plant acceptance when one cultivar was exposed to volatiles of the other and vice versa (two-way interaction). In the specific cultivar pair where Apolon was exposed to Enola (Bulgaria), there was a significant reduction in the number of aphids settled on Apolon plants (“one-way” interaction). The remaining cultivar pairs showed no reduction in aphid plant acceptance (**Table 2**).

**Table 2:** The mean (± SE) number of *Rhopalosiphum padi* aphids that settled on winter wheat cultivars (receivers) when exposed to volatiles from a paired ‘emitter’ cultivar, compared to the same receiver cultivar exposed to clean air.

Country of origin	Emitter cultivar	Receiver cultivar	Aphids on receiver	Aphids on control	P-value		Response type
Bosnia and Herzegovina & Serbia	NS Rani otkos	NS 40S	7.37 ± 0.17	8.63 ± 0.15	**0.0001**	***	*Two-way interaction*
	NS 40S	NS Rani otkos	7.21 ± 0.18	8.7 ± 0.15	**0.0001**	***	
	NS Rani otkos	Simonida	8.1 ± 0.16	8.61 ± 0.16	0.13		*No interaction*
	Simonida	NS Rani otkos	8.6 ± 0.17	8.7 ± 0.15	0.97		
	NS 40s	Simonida	8.15 ± 0.17	8.61 ± 0.16	0.20		*No interaction*
	Simonida	NS 40S	8.22 ± 0.15	8.63 ± 0.15	0.35		
Bulgaria	Enola	Lazarka	7.75 ± 0.14	9.1 ± 0.11	**0.0001**	***	*Two-way interaction*
	Lazarka	Enola	7.65 ± 0.24	8.7 ± 0.12	**0.0004**	***	
	Enola	Apolon	8.0 ± 0.19	8.7 ± 0.11	**0.036**	*	*One-way interaction*
	Apolon	Enola	8.05 ± 0.15	8.7 ± 0.12	0.056		
	Lazarka	Apolon	8.9 ± 0.13	8.7 ± 0.11	0.90		*No interaction*
	Apolon	Lazarka	8.95 ± 0.09	9.1 ± 0.11	0.98		
United Kingdom	Hereward	Xi19	7.0 ± 0.19	8.95 ± 0.20	**0.0001**	***	*Two-way interaction*
	Xi19	Hereward	8.15 ± 0.17	8.95 ± 0.14	**0.014**	*	
	Claire	Hereward	7.8 ± 0.16	8.95 ± 0.14	**0.0002**	***	*Two-way interaction*
	Hereward	Claire	7.45 ± 0.14	9.0 ± 0.15	**0.001**	***	
	Xi19	Claire	8.4 ± 0.11	8.9 ± 0.18	0.19		*No interaction*
	Claire	Xi19	8.15 ± 0.25	7.8 ± 0.33	0.84		

Wheat cultivars originated from different countries. Significant differences are highlighted in bold (* = P-value< 0.05, ** = P-value< 0.01, *** = P-value< 0.001). We categorized responses into three types: No interaction occurred when the numbers of aphids settled on control plants did not differ from those on receiver plants exposed to volatiles from another cultivar. One-way interaction was indicated by significant reduction in aphid acceptance on the receiver exposed to volatiles from a specific emitter cultivar compared to control. Two-way interaction was characterized by a reduction in aphid acceptance on receiver cultivars compared with control, regardless of the emitter, indicating volatile interaction occurred in both directions.

In the spring barley cultivars, the pairs, Vertti+Toria (Finland) and Fairytale+Salome (Sweden) exhibited “two-way” interaction while Jaran exposed to Matej, and Jaran exposed to Scarlett (Bosnia and Herzegovina), and Anakin exposed to Salome (Sweden) demonstrated “one-way” interaction (**Table 3**) all resulting in significantly reduced aphid plant acceptance. The other pairs did not affect aphid plant acceptance indicating “no-interaction”.

**Table 3:** The mean (± SE) number of *Rhopalosiphum padi* aphids that settled on spring barley cultivars after exposure to the volatiles from another cultivar.

Country of origin	Emitter cultivar	Receiver cultivar	Aphids on receiver	Aphids on control	P-value		Response type
Bosnia and Herzegovina	Matej	Jaran	5.00 ± 0.39	6.60 ± 0.42	**0.021**	*	*One-way interaction*
	Jaran	Matej	4.3 ± 0.41	4.3 ± 0.42	0.99		
	Scarlett	Jaran	5.05 ± 0.38	6.60 ± 0.42	**0.025**	*	*One-way interaction*
	Jaran	Scarlett	6.10 ± 0.31	5.80 ± 0.48	0.96		
	Scarlett	Matej	3.60 ± 0.51	4.30 ± 0.42	0.76		*No interaction*
	Matej	Scarlett	5.80 ± 0.33	5.80 ± 0.48	0.99		
Finland	Vertti	Toria	7.11 ± 0.25	8.17 ± 0.20	**0.01**	**	*Two-way interaction*
	Toria	Vertti	5.44 ± 0.22	6.61 ± 0.32	**0.01**	**	
	Vertti	Alvari	6.39 ± 0.29	7.17 ± 0.31	0.25		*No interaction*
	Alvari	Vertti	7.22 ± 0.17	6.61 ± 0.32	0.42		
	Toria	Alvari	7.11 ± 0.23	7.17 ± 0.31	0.99		*No interaction*
	Alvari	Toria	7.28 ± 0.24	8.17 ± 0.20	0.56		
Sweden	Fairytale	Salome	7.25 ± 0.41	8.5 ± 0.26	**0.027**	*	*Two-way interaction*
	Salome	Fairytale	6.75 ± 0.37	8.05 ± 0.28	**0.023**	*	
	Anakin	Salome	8.0 ± 0.37	8.5 ± 0.26	0.59		*One-way interaction*
	Salome	Anakin	6.7 ± 0.45	8.35 ± 0.18	**0.018**	*	
	Fairytale	Anakin	7.1 ± 0.35	8.35 ± 0.18	0.11		*No interaction*
	Anakin	Fairytale	8.06 ± 0.36	8.05 ± 0.28	0.89		

Significant differences are highlight in bold (* = P-value< 0.05, ** = P-value< 0.01, *** = P-value< 0.001). Cultivars originated from different countries. We categorized responses into three types: No interaction inferred when the numbers of aphids settled on control plants did not differ from those exposed to volatiles from another cultivar. One-way interaction was indicated by significant reduction in aphid acceptance on the receiver exposed to volatiles from a specific emitter cultivar compared to the control. Two-way interaction was characterized by a reduction in aphid acceptance on receiver cultivars, regardless of the emitter, indicating volatile interaction occurred in both directions.

### Effect of cultivar mixtures on insects and plant traits in field trials

The composition of the aphid community varied between countries and sampling occasions (**Supplementary Table S1**).

**Figure 1:**
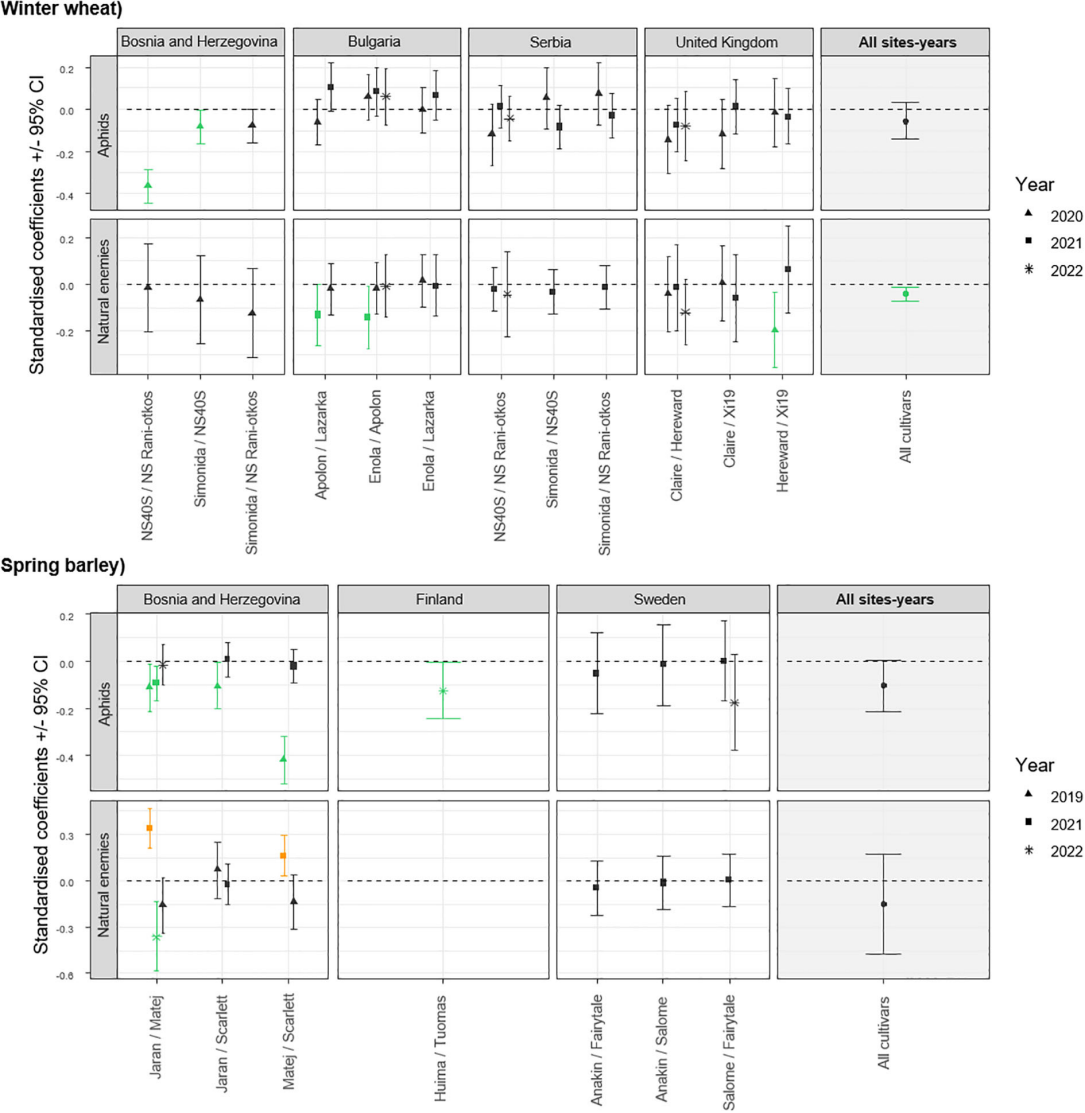
Standardised coefficient parameters (± 95% confidence interval) extracted from models testing the effect of cultivar mixtures on the abundance of aphids and their natural enemies, by comparing values observed in plots with a mixture of cultivars to values obtained from an expected mixtures based on monoculture plots. Data were collected in 16 field trials in Bosnia and Herzegovina, Bulgaria, Finland, United Kingdom, Serbia, and Sweden. Cultivars tested were either winter wheat or spring barley. Coefficients are presented for each pair of cultivars tested, different for each measurement done in the field. Results from a meta-analysis aggregating the results from the different sites are also represented on the right side (All sites-years). Positive values indicate that the measurement is higher in the cultivar mixture compared to monoculture, and negative values the opposite. Orange symbols represent model coefficients that are significantly positive (their confidence interval does not exceed 0), while the green symbols represent model coefficients that are significantly negative.

The screening method conducted under controlled conditions showed that the overall effects of volatile interaction between cultivars on aphid infestation may not be straightforward. Of the 86 cultivar pairs tested in the laboratory, 10 demonstrated efficacy in reducing aphid plant acceptance, but only 6 pairs exhibited ‘two-way interaction’. When analyzing each pair independently across different trials, significant effects were observed in specific instances. In particular, in Bosnia and Herzegovina, significant reductions were observed for the pairs NS40S+NS Rani otkos, and Simonida+NS40S in winter wheat in 2020 (**Figure 1** and **Supplementary Figure S4**). A similar trend was observed in the spring barley pairs in Bosnia and Herzegovina, with significant reduction in aphid infestation for the combinations Jaran+Matej in 2019 and 2021, Jaran+Scarlett in 2019, and Matej+Scarlett in 2019 as well as in 2022 in Finland for the combination of cultivars Huima+Tuomas (**Figure 1** and **Supplementary Figure S4**).

The abundance of natural enemies was significantly reduced in Bulgaria in the winter wheat pairs Apolon+Lazarka and Enola+Lazarka in 2021, and in the United kingdom in the Hereward+Xi19 mixture in 2020 (**Figure 1** and **Supplementary Figure S5**). Similar effects of cultivar mixtures on the natural enemy abundance in spring barley were observed in Bosnia and Herzegovina for the Jaran+Matej mixtures in 2022 compared to their monoculture plots (**Figure 1** and **Supplementary Figure S5**). Conversely, an increase in the natural enemy abundance was observed in 2021 for the Jaran+Matej and Matej+Scarlett spring barley mixtures of in Bosnia and Herzegovina (**Figure 1** and **Supplementary Figure S5**).

**Figure 2:**
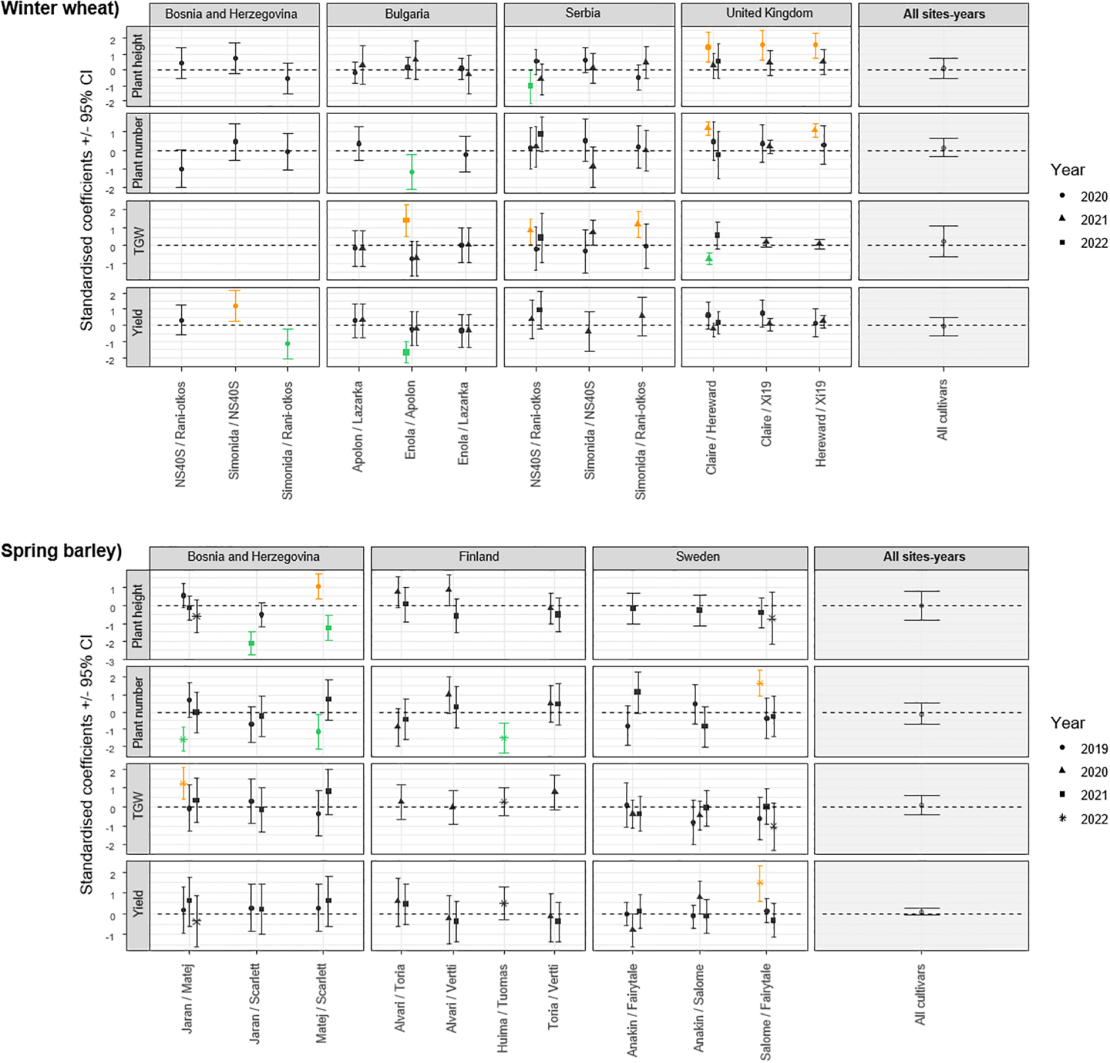
Standardised coefficient parameters (± 95% confidence interval) extracted from models testing the effect of cultivar mixtures on plant height, plant number, thousand grain weight, and yield, by comparing values observed in plots with a mixture of cultivars to values obtained from an expected mixtures based on monoculture plots. Data were collected in 20 field trials in Bosnia and Herzegovina, Bulgaria, Finland, United Kingdom, Serbia, and Sweden. Cultivars tested were either winter wheat or spring barley. Coefficients are presented for each pair of cultivars tested, different for each measurement done in the field. Results from a meta-analysis aggregating the results from the different sites are also represented on the right side (All sites-years). Positive values indicate that the measurement is higher in the cultivar mixture compared to monoculture, and negative values the opposite. Orange symbols represent model coefficients that are significantly positive (their confidence interval does not exceed 0), while the green symbols represent model coefficients that are significantly negative.

The impact of cultivar mixtures on plant traits was also evident in specific combinations, highlighting the importance of strategic cultivar selection in optimizing agricultural outcomes. For instance, a significant increase in plant height was observed for all winter wheat cultivar mixtures in United Kingdom in 2020 (**Figure 2**) and for the Matej+Scarlett barley mixture in Bosnia and Herzegovina in 2019 compared to their theoretically expected mixtures (**Figure 2**). A significant decrease in plant height was observed in Serbia in 2022 for the NS40S+NS Rani otkos mixture (**Figure 2**) and Bosnia and Herzegovina in 2021 for the Jaran+Scarlett and Matej+Scarlett mixtures (**Figure 2**). However, significant reductions in the number of plants per 1-meter were found in winter wheat mixtures of Enola+Apolon in Bulgaria in 2020 (**Figure 2**), and in spring barley mixtures of Matej+Scarlett in 2019 and Jaran+Matej in 2022 in Bosnia and Herzegovina, and in Huima+Tuomas mixture in 2022 in Finland (**Figure 2**).

Significant increases in TGW were observed in winter wheat mixtures in Bulgaria for the Enola+Apolon in 2022, and in Serbia for the mixtures of Simonida+NS Rani otkos and NS40S+NS Rani otkos in 2021 (**Figure 2**) and for the spring barley mixture of Jaran+Matej in Bosnia and Herzegovina in 2022 (**Figure 2**), when compared to their respective monocultures. Decrease in TGW was recorded only in United Kingdom in 2021 for the Claire+Hereward mixture (**Figure 2**). Yield increase was observed in spring barley in Bosnia and Herzegovina in 2020 for the Simonida+NS40S mixture (**Figure 2**) and in Sweden in 2022 for the Anakin+Salome mixture (**Figure 2**). Conversely, yield decreases were recorded in Bosnia and Herzegovina in 2020 for the Simonida+NS Rani otkos mixture and in Bulgaria in 2022 for the Enola+Apolon mixture (**Figure 2**).

### Relationship between screening of aphid plant acceptance in the laboratory and aphid population development in field trials

**Figure 3:**
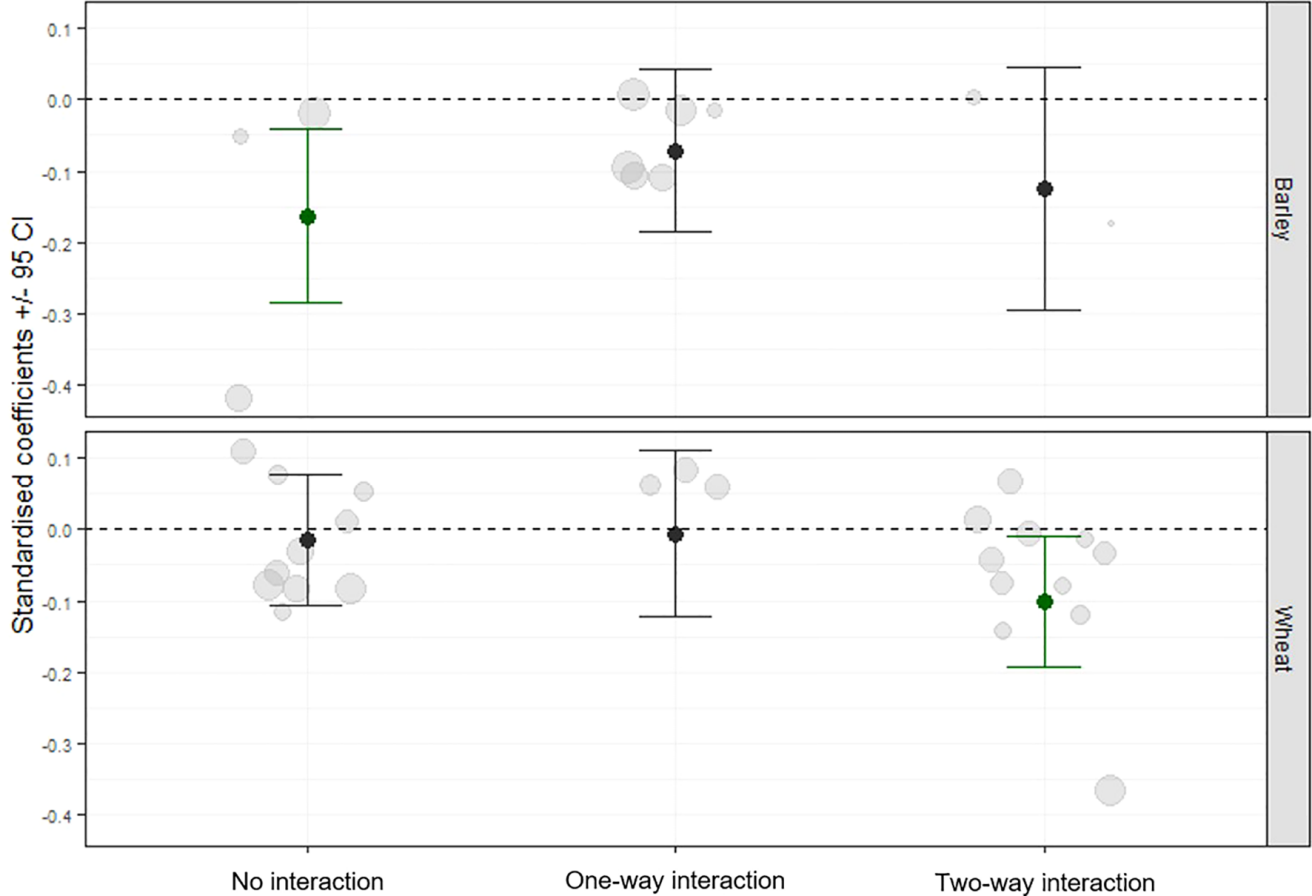
Standardised coefficient parameters (± 95% confidence intervals) (black dots) were extracted from meta-analysis models that tested the effect of volatile interaction between cultivars on aphids. The models compared values observed in plots with a mixture of cultivars to values obtained from an expected mixtures based on monoculture plots. Data were collected from 16 field trials conducted in Bosnia and Herzegovina, Bulgaria, Finland, United Kingdom, Serbia, and Sweden. Grey dots represent the effect size used in the model. The size of the grey dots is related to the precision (1/SE) of the model coefficients used for the meta-analysis. The cultivars were divided into pairs based on the outcomes of an aphid plant acceptance test conducted in controlled conditions and the observed interaction between the cultivars via volatiles, which induced defence mechanisms (no interaction, one-way interaction, two-way interaction).

A significant reduction in aphid infestation in the field was observed in cultivar combinations that exhibited “two-way interaction” under controlled laboratory conditions (QM = 20.62, df = 2, p< 0.0001) (**Figure 3**). In contrast, cultivar pairs classified as having “no interaction” or “one-way interaction” showed no significant effect on aphid infestation.

## Discussion

Our study is the first to demonstrate that screening for defense priming through constitutively released VOCs in controlled environments can yield consistent results in the field on aphid suppression in some countries. Despite the variation in performance, cultivar pairs identified as aphid suppressive under controlled conditions showed potential benefits for aphid management in the field. In controlled laboratory studies, we demonstrated that some wheat or barley cultivars were less accepted by cereal aphids when exposed to the volatiles emitted by a specific cultivar. Notably, with some cultivars we observed a bidirectional plant response to neighbor VOCs, whereby one cultivar primed defense in another cultivar and vice versa. However, in some pairs, only the VOCs released from a specific cultivar primed defense response in another cultivar, making it less acceptable to aphids. Cultivar pairs that demonstrated bidirectional interaction under controlled conditions tended experience lower incidence of aphid infestation in the field compared to monocultures. This relationship between two-way interaction via VOCs and reduced aphid populations suggests that cultivar pairs exhibiting reciprocal priming effects can be more resilient to aphid pressure. Recent studies further support the potential of VOC interactions as a predictor of field performance, underscoring the importance of selecting cultivar pairs based on their ability to engage in effective VOC signaling (Kheam et al., 2023, 2024a). Our findings suggest that interaction between specific cultivars via constitutively released VOCs may serve as a predictor of the aphid’s performance when these cultivars are paired in the field. Therefore, we propose a new method to increase intra-specific diversity in crops by pairing cultivars in mixtures based on their capacity to prime neighbor defense mechanisms via constitutively released VOCs.

The reduction in aphid infestation in the field was significant in cultivar mixtures in Bosnia and Herzegovina and Finland, while in other trials the effects were variable, with a tendency towards reduction. These results are particularly encouraging as they show that the positive effects of increased cultivar diversity on pest suppression can be maintained under realistic growing conditions. The observed variation between cultivar pairs and countries may be attributed to the relatively low abundance of aphids in several trials, which may have limited their ability to detect a difference between treatments. Insect responses to plant VOCs often differ between controlled and natural environments due to factors like wind, temperature, and community complexity, which influence VOCs dispersal and perception (Schuman and Baldwin, 2018; Schuman, 2023). For instance, transgenic wheat producing (E)-β-farnesene, an aphid alarm pheromone, effectively repelled aphids and attracted their natural enemies in laboratory settings, but showed no significant impact on aphid populations in field trials. This highlights how aphid responses to plant VOCs vary between controlled and natural environments due to ecological complexity (Bruce et al., 2015). Nevertheless, relying exclusively on screening under controlled conditions to predict pest abundance in cultivar mixtures may fail to consider other crucial environmental factors that shape plant-plant and plant-insect interactions in the field. Consequently, a multi-year field study encompassing diverse aphid densities and environmental conditions is essential to comprehensively capture the impact of these complex interactions in selected cultivar mixtures and their effects on aphid performance.

Previous field studies have demonstrated a significant reduction in aphid infestation in cultivar mixtures compared to monoculture (Dahlin et al., 2018; Tous-Fandos et al., 2024). Nevertheless, an increase in intra-specific plant diversity does not necessarily result in reduction in herbivore pressure, highlighting the importance of selecting the “right kind of diversity” (Shoffner and Tooker, 2013). A recent study has shown that volatiles from one barley cultivar can induce defense responses in another cultivar, affecting feeding behavior and performance of *R. padi*. This response in a receiving cultivar depended on the genetic identity of the emitting cultivar (Kheam et al., 2023). Therefore, optimization of the complementarity between cultivars in terms of their volatile interactions may serve to enhance the reliability of this diversification approach for the purpose of improved pest control. Our field trials revealed considerable variability in the impact of cultivar mixtures on aphid infestation, which was dependent on the specific cultivars involved and the local environmental conditions that influenced the type of interactions between cultivars, or between cultivars in mixtures and other organisms. It has been demonstrated that environmental stressors can significantly impact the production and emission of VOCs in plants, leading to alternations in their physiological and biochemical processes (Midzi et al., 2022). During our field trials, severe drought conditions in 2020 and 2021 likely affected plant growth, volatile emissions and interactions with aphids and their natural enemies, potentially affecting the overall results of the study. Such modifications in VOC composition have the potential to influence plant-plant interaction by modifying essential signaling mechanisms involved in defense responses (Peñuelas and Staudt, 2010). These findings underscore the importance of optimizing cultivar combinations for specific environments, as some VOC interactions may be more sensitive to local climatic and soil conditions than others.

The cultivar mixtures did not significantly affect the abundance of aphid natural enemies in our field trials. This may be attributed to the relatively low aphid abundance in the majority of the experiments. It is important to note that our observations of the presence of natural enemies were focused on the period of intensive prey search, subsequent to the colonization of the field by aphids, rather than during the habitat search phase. This timing may, at least in part, explain the lack of observed effects on the occurrence of natural enemies (Ninkovic and Pettersson, 2003). Cultivar mixtures led to variable increases in predators like *Harmonia axyridis*, due to habitat complexity and prey availability, with these effects exhibited variations across seasons (Grettenberger and Tooker, 2020). A recent study has demonstrated that soybean cultivar mixtures significantly increase the abundance of natural enemies such as ladybirds, parasitoid wasps, and dragonflies, at specific plant stages due to enhanced microhabitat diversity and volatile interactions between plants (Kheam et al., 2024b). Natural enemies do exploit changes in plant emitted VOCs when searching for prey (Clavijo McCormick et al., 2012), and this may be an underlying mechanism that influences their behavior when shifting from habitat to prey search, regardless of cultivar mixture. Furthermore, the mobility of ladybirds (Sloggett, 2021) and hoverflies (Francuski et al., 2013) present a challenge to accurate monitoring. The activity of these insects fluctuates significantly in response to weather conditions, which makes it challenging to detect any stable effects of cultivar mixtures on their abundance. Additionally, the relatively small plot sizes used in these trials may have influenced the abundance of natural enemies, which are highly mobile and may be more closely related to prey availability than to the specific cultivar mixture treatments. Some studies suggest that complex landscapes are associated with higher predator populations than simpler ones, which could further bolster the pest-suppressive effects of cultivar mixtures (Ali et al., 2020; Haan et al., 2020). Though cultivar mixtures showed minimal impact on natural enemy populations, their resilience and yield under adverse conditions support their potential for sustainable crop management. This suggests that while cultivar diversity benefits plant-insect interactions, larger scale or habitat diversification may be needed to bolster natural enemy populations effectively.

We did not observe a clear and consistent effect of the cultivar mixtures on the number of plants, plant height, TGW and crop yield. However, the yield of cultivar mixtures is not always greater than that of monocultures (Grettenberger and Tooker, 2020). The interactions between different cultivar combinations and varying environmental conditions can significantly affect the yield of these mixtures (Knapp and van der Heijden, 2018). The results of this study suggest that selecting cultivar pairs based on their ability to interact via VOCs can improve agroecosystem functioning. These specific cultivar mixtures emerged as a potent ecological strategy to mitigate the adverse effects of local growing conditions and improve productivity, stability and resilience of the agroecosystem. This validates the approach as a valuable tool for identifying the optimal cultivar pairings that maximize the benefits of intra-specific diversity.

## Conclusion

Screening of cereal cultivars for their capacity to interact via VOCs under controlled conditions is a promising approach to rapidly identify a large number of cultivars suitable for the design of pest suppressing mixtures. This method highlights the potential of integrating chemical ecology into the design of cultivar mixtures to improve productivity and resilience in agricultural systems. However, it is essential to optimize this approach to identify cultivar pairs with high mutual compatibility. Further experiments should also consider environmental factors, cultivar-specific traits such as phenological complementarity, pathogen susceptibility, and grain quality to ensure that these mixtures can be effectively adapted and commercialized by farmers. Future research should investigate the mechanisms behind these interactions and explore their applicability in diverse agricultural settings.

## Data Availability

The raw data supporting the conclusions of this article will be made available by the authors, without undue reservation.
