# Supplementary material for: Volatile-mediated plant interactions: an innovative approach to cultivar mixture selection for enhanced pest resilience
Source: Front Plant Sci. 2025 Apr 8;16:1550678. doi: 10.3389/fpls.2025.1550678 (PMC12011781; doi:10.3389/fpls.2025.1550678)
Supplement: Supplementary file 1 [file Table1.docx]

**Volatile-Mediated Plant Interactions: An Innovative Approach to Cultivar Mixture Selection for Enhanced Pest Resilience**

Dimitrije Markovic, Gaëtan Seimandi-Corda, Vili Harizanova, Atanaska Stoeva, Sari Himanen, Stephanie Saussure, Andja Radonjic, Gordana Djuric, Ivana Lalicevic, Sokha Kheam, Merlin Rensing, Jannicke Gallinger, Samantha M. Cook and Velemir Ninkovic


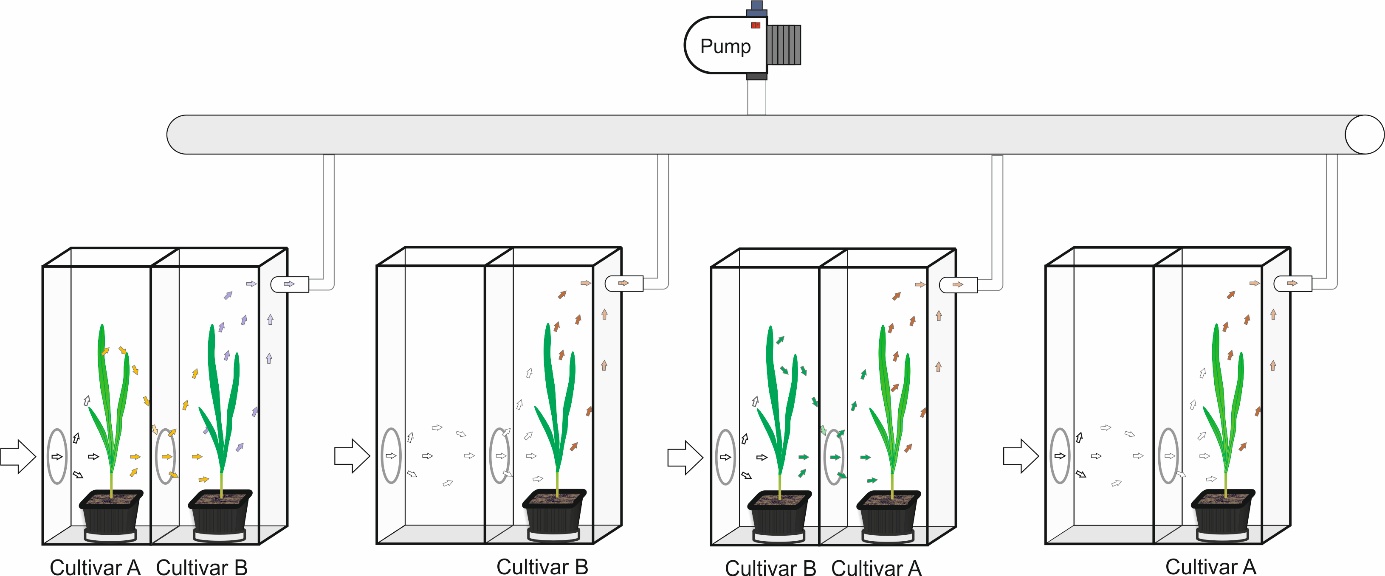


Figure S1. Graphical illustration of the exposure system in which one cultivar was exposed to the volatiles from another one. A receiving cultivar that had been exposed to clean air was utilised as a control.
