# Supplementary material for: Volatile-mediated plant interactions: an innovative approach to cultivar mixture selection for enhanced pest resilience
Source: Front Plant Sci. 2025 Apr 8;16:1550678. doi: 10.3389/fpls.2025.1550678 (PMC12011781; doi:10.3389/fpls.2025.1550678)
Supplement: Supplementary file 2 [file Table2.docx]

**Volatile-Mediated Plant Interactions: An Innovative Approach to Cultivar Mixture Selection for Enhanced Pest Resilience**

Dimitrije Markovic, Gaëtan Seimandi-Corda, Vili Harizanova, Atanaska Stoeva, Sari Himanen, Stephanie Saussure, Andja Radonjic, Gordana Djuric, Ivana Lalicevic, Sokha Kheam, Merlin Rensing, Jannicke Gallinger, Samantha M. Cook and Velemir Ninkovic


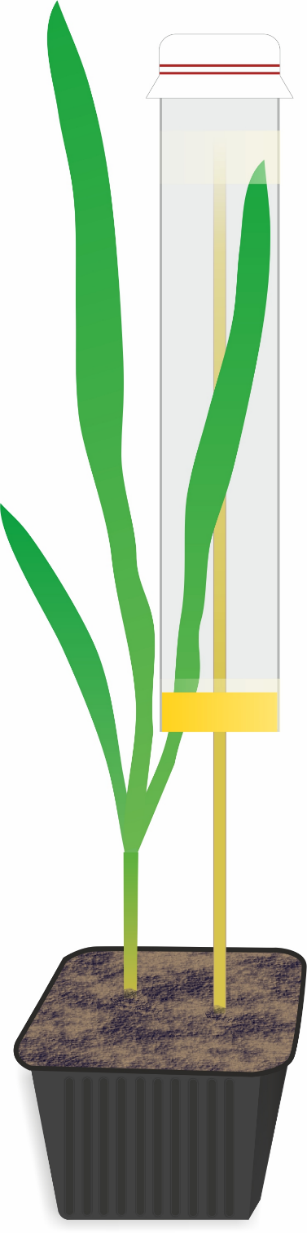


Figure S2. Aphid plant acceptance test setup. The bioassay chamber consisted of 100-ml polystyrene tube (2.5 cm x 25cm). The second leaf of a single ‘receiver’ plant was inserted into one end of the tube through a slit made in a plastic sponge that plugged the tube’s bottom end, without detaching the leaf from the main plant. Ten wingless *Rhopalosiphum padi* were placed in the top of the tube, which was then sealed with nylon netting to prevent their escape. To prevent mechanical damage to the plant, the bioassay tube was attached to a wooden stick to provide support.
