# Supplementary material for: Volatile-mediated plant interactions: an innovative approach to cultivar mixture selection for enhanced pest resilience
Source: Front Plant Sci. 2025 Apr 8;16:1550678. doi: 10.3389/fpls.2025.1550678 (PMC12011781; doi:10.3389/fpls.2025.1550678)
Supplement: Supplementary file 3 [file Table3.docx]

**Volatile-Mediated Plant Interactions: An Innovative Approach to Cultivar Mixture Selection for Enhanced Pest Resilience**

Dimitrije Markovic, Gaëtan Seimandi-Corda, Vili Harizanova, Atanaska Stoeva, Sari Himanen, Stephanie Saussure, Andja Radonjic, Gordana Djuric, Ivana Lalicevic, Sokha Kheam, Merlin Rensing, Jannicke Gallinger, Samantha M. Cook and Velemir Ninkovic

Figure S3. Example of calculation of the expected cultivar mixture values based on plot position according to rows and columns.
