# Supplementary material for: Volatile-mediated plant interactions: an innovative approach to cultivar mixture selection for enhanced pest resilience
Source: Front Plant Sci. 2025 Apr 8;16:1550678. doi: 10.3389/fpls.2025.1550678 (PMC12011781; doi:10.3389/fpls.2025.1550678)
Supplement: Supplementary file 4 [file Table4.docx]

**Volatile-Mediated Plant Interactions: An Innovative Approach to Cultivar Mixture Selection for Enhanced Pest Resilience**

Dimitrije Markovic, Gaëtan Seimandi-Corda, Vili Harizanova, Atanaska Stoeva, Sari Himanen, Stephanie Saussure, Andja Radonjic, Gordana Djuric, Ivana Lalicevic, Sokha Kheam, Merlin Rensing, Jannicke Gallinger, Samantha M. Cook and Velemir Ninkovic


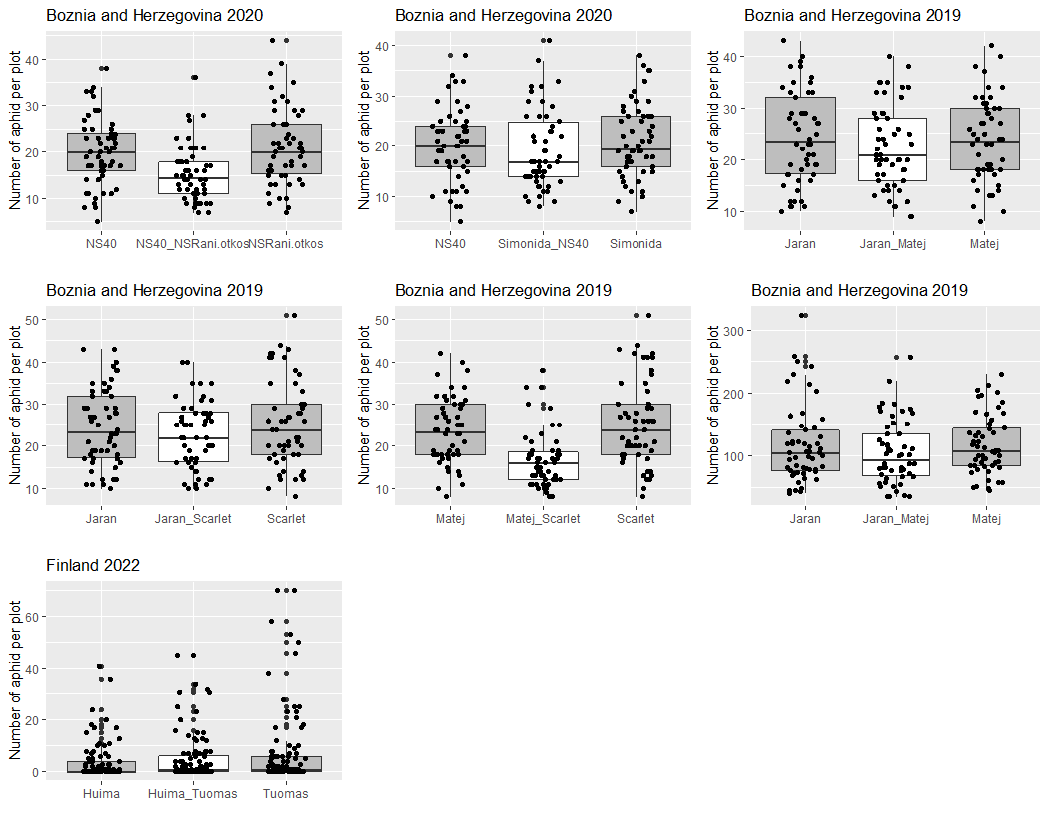


Figure S4. Boxplots (25-75 percentiles and median) of the number of aphids per plot per sampling occasion in different treatments (one cultivar or mixtures of cultivars) of different field trials where a significant effect of the cultivar mixture was observed. Black dots represent the raw data of the number of aphids per plot.
