# Supplementary material for: Volatile-mediated plant interactions: an innovative approach to cultivar mixture selection for enhanced pest resilience
Source: Front Plant Sci. 2025 Apr 8;16:1550678. doi: 10.3389/fpls.2025.1550678 (PMC12011781; doi:10.3389/fpls.2025.1550678)
Supplement: Supplementary file 5 [file Table5.docx]

**Volatile-Mediated Plant Interactions: An Innovative Approach to Cultivar Mixture Selection for Enhanced Pest Resilience**

Dimitrije Markovic, Gaëtan Seimandi-Corda, Vili Harizanova, Atanaska Stoeva, Sari Himanen, Stephanie Saussure, Andja Radonjic, Gordana Djuric, Ivana Lalicevic, Sokha Kheam, Merlin Rensing, Jannicke Gallinger, Samantha M. Cook and Velemir Ninkovic


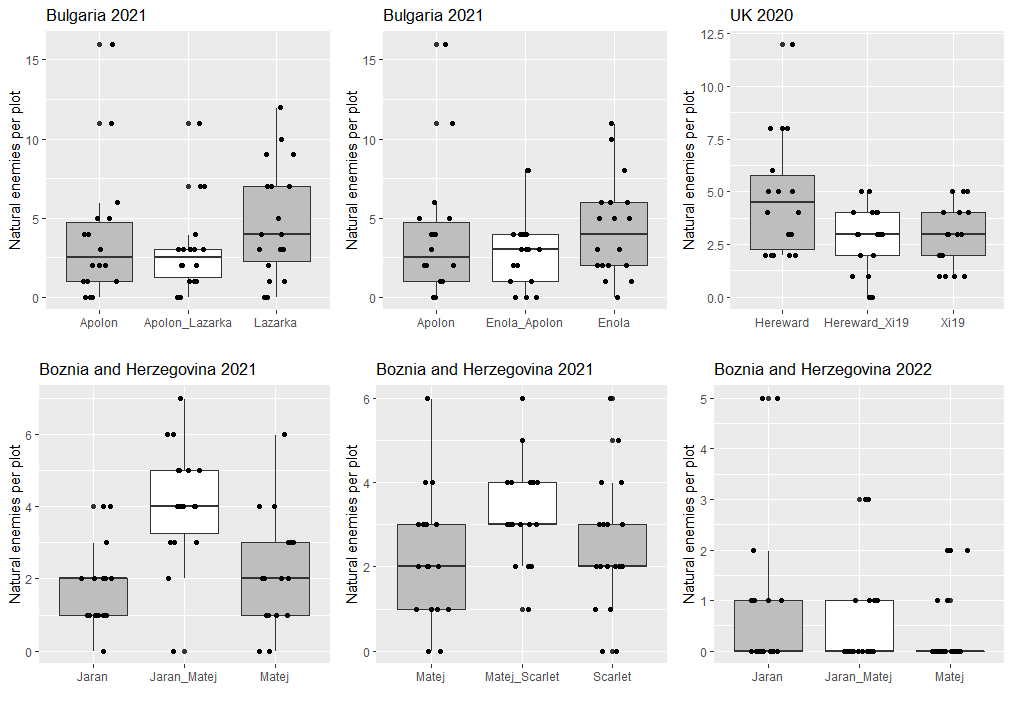


Figure S5. Boxplots (25-75 percentiles and median) of the number of natural enemies (ladybirds and hoverflies) per plot per sampling occasion in different treatments (one cultivar or mixtures of cultivars) of different field trials where a significant effect of the cultivar mixture was observed. Black dots represent the raw data of the number of natural enemies per plot.
