# Supplementary material for: Volatile-mediated plant interactions: an innovative approach to cultivar mixture selection for enhanced pest resilience
Source: Front Plant Sci. 2025 Apr 8;16:1550678. doi: 10.3389/fpls.2025.1550678 (PMC12011781; doi:10.3389/fpls.2025.1550678)
Supplement: Supplementary file 6 [file Table6.docx]

**Volatile-Mediated Plant Interactions: An Innovative Approach to Cultivar Mixture Selection for Enhanced Pest Resilience**

Dimitrije Markovic, Gaëtan Seimandi-Corda, Vili Harizanova, Atanaska Stoeva, Sari Himanen, Stephanie Saussure, Andja Radonjic, Gordana Đurić, Ivana Lalicević, Sokha Kheam, Merlin Rensing, Jannicke Gallinger, Samantha M. Cook and Velemir Ninkovic

Table S1. Proportion of the three cereal aphid species found in the field trials completed in six countries (2019-2022).

| **Country** | **Season** | ***Sitobion avenae*** | ***Rhopalosiphum padi*** | ***Metopolophium dirhodum*** |
| --- | --- | --- | --- | --- |
| Bosnia and Herzegovina | 2019 | 100% | 0% | 0% |
|  | 2020 | 100% | 0% | 0% |
|  | 2021 | 100% | 0% | 0% |
|  | 2022 | 100% | 0% | 0% |
| Bulgaria | 2020 | 100% | 0% | 0% |
|  | 2021 | 100% | 0% | 0% |
|  | 2022 | 100% | 0% | 0% |
| Finland | 2022 | 96% | 1% | 3% |
| Serbia | 2020 | 100% | 0% | 0% |
|  | 2021 | 100% | 0% | 0% |
|  | 2022 | 100% | 0% | 0% |
| Sweden | 2021 | 0% | 100% | 0% |
|  | 2022 | 96% | 4% | 0% |
| United Kingdom | 2020 | 56% | 23% | 21% |
|  | 2021 | 16% | 34% | 50% |
|  | 2022 | 77% | 9% | 14% |
